# Virtual screening of anti-HIV1 compounds against SARS-CoV-2: machine learning modeling, chemoinformatics and molecular dynamics simulation based analysis

**DOI:** 10.1038/s41598-020-77524-x

**Published:** 2020-11-23

**Authors:** Mahesha Nand, Priyanka Maiti, Tushar Joshi, Subhash Chandra, Veena Pande, Jagdish Chandra Kuniyal, Muthannan Andavar Ramakrishnan

**Affiliations:** 1 Environmental Information System on Himalayan Ecology, G.B. Pant National Institute of Himalayan Environment, Kosi-Katarmal, Almora, Uttarakhand 263 643 India; 2 Centre for Environmental Assessment and Climate Change, G.B. Pant National Institute of Himalayan Environment, Kosi-Katarmal, Almora, Uttarakhand 263 643 India; 3 Department of Biotechnology, Kumaun University, Bhimtal Campus, Bhimtal, Uttarakhand 263 136 India; 4 Department of Botany, Kumaun University, S.S.J. Campus, Almora, Uttarakhand 263 601 India; 5 ICAR-Indian Veterinary Research Institute, Bengaluru, Karnataka 560 024 India

**Keywords:** Virtual screening, High-throughput screening

## Abstract

COVID-19 caused by the SARS-CoV-2 is a current global challenge and urgent discovery of potential drugs to combat this pandemic is a need of the hour. 3-chymotrypsin-like cysteine protease (3CLpro) enzyme is the vital molecular target against the SARS-CoV-2. Therefore, in the present study, 1528 anti-HIV1compounds were screened by sequence alignment between 3CLpro of SARS-CoV-2 and avian infectious bronchitis virus (avian coronavirus) followed by machine learning predictive model, drug-likeness screening and molecular docking, which resulted in 41 screened compounds. These 41 compounds were re-screened by deep learning model constructed considering the IC_50_ values of known inhibitors which resulted in 22 hit compounds. Further, screening was done by structural activity relationship mapping which resulted in two structural clefts. Thereafter, functional group analysis was also done, where cluster 2 showed the presence of several essential functional groups having pharmacological importance. In the final stage, Cluster 2 compounds were re-docked with four different PDB structures of 3CLpro, and their depth interaction profile was analyzed followed by molecular dynamics simulation at 100 ns. Conclusively, 2 out of 1528 compounds were screened as potential hits against 3CLpro which could be further treated as an excellent drug against SARS-CoV-2.

## Introduction

Severe Acute Respiratory Syndrome Coronavirus 2 (SARS-CoV-2) is the causative agent of the COVID-19. According to the *Coronaviridae* Study Group (CSG) of the International Committee on Taxonomy of Viruses (ICTV), the SARS-CoV-2 is classified under the species severe acute respiratory syndrome-related coronavirus, genus *Betacoronavirus*, family *Coronaviridae*, suborder *Cornidovirineae*, order *Nidovirales*, and realm *Riboviria*^1^. Some other human respiratory coronaviruses include HCoV-229E, -NL63, -OC43, and -HKU1 which are common. On the other hand, coronaviruses viz., SARS-CoV, MERS-CoV, SARS-CoV-2 are deadly^2^. Two, vital drug targets of SARS-CoV-2 are spike (S) protein which binds with the Angiotensin-Converting Enzyme 2 (ACE2) receptor of humans and facilitates the viral entry in the host cell and 3CLpro which involved in the replication process of the virus^3^.

SARS-CoV-2, a single-stranded positive-sense RNA virus and belongs to the beta-coronaviruses group that produces a transcription product of ∼800 kDa peptide. This polypeptide is proteolytically processed by papain-like protease (PLpro) and 3-chymotrypsin-like protease (3CLpro). 3CLpro cleaves at 11 sites and produce various non-structural proteins (nsp) that are necessary for viral replication^4^. Thus, blocking viral replication by inhibiting 3CLpro is one of the key strategies in the drug development process. Some drugs in this aspect include ASC09 or darunavir/cobicistat, which inhibit the 3C-like protease (3CLpro)^5^. In the CAS REGISTRY, the highest number of patents and potential drug candidates have been registered against 3CLpro among all the target proteins of SARS-CoV-2, which directly reflect its potentiality as a drug target^6^.

**Figure 1:**
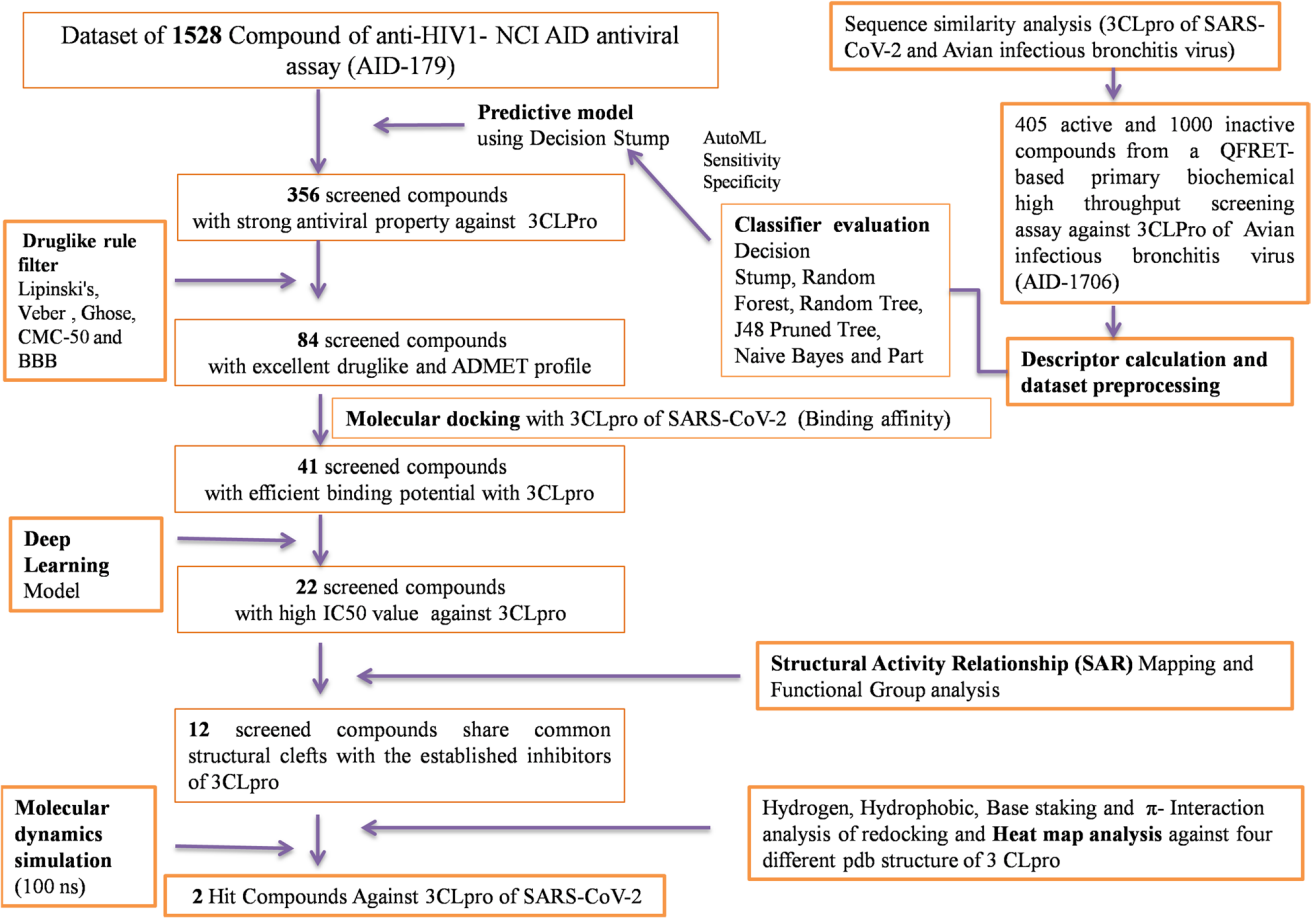
Detailed outline of the study.

Both SARS-CoV-2 and HIV-1 are single-stranded RNA viruses (+ ssRNA) viruses^7,8^. Nowadays, several anti-HIV drugs including darunavir, cobicistat, and ASC09F have been considered for clinical trials against SARS-CoV-2 infection^9^. Anti -HIV inhibitors like indinavir and darunavir also have been reported to have excellent binding potentials with SARS-CoV-2 3CLpro^10^. Further, HIV protease inhibitors showed antiviral activity against many viruses including SARS-CoV^11–14^. Therefore, the current study aims to screen 1528 anti HIV1 compounds against 3CLpro by applying several in silico tools, viz., predictive modeling, ADMET and drug-likeness screening, multiple sequence alignment, molecular docking, deep learning, chemical space mapping and molecular dynamics simulation (Fig. 1).

## Results

### Sequence similarity analysis, screening by the predictive model, dug-likeness and ADMET

**Figure 2:**
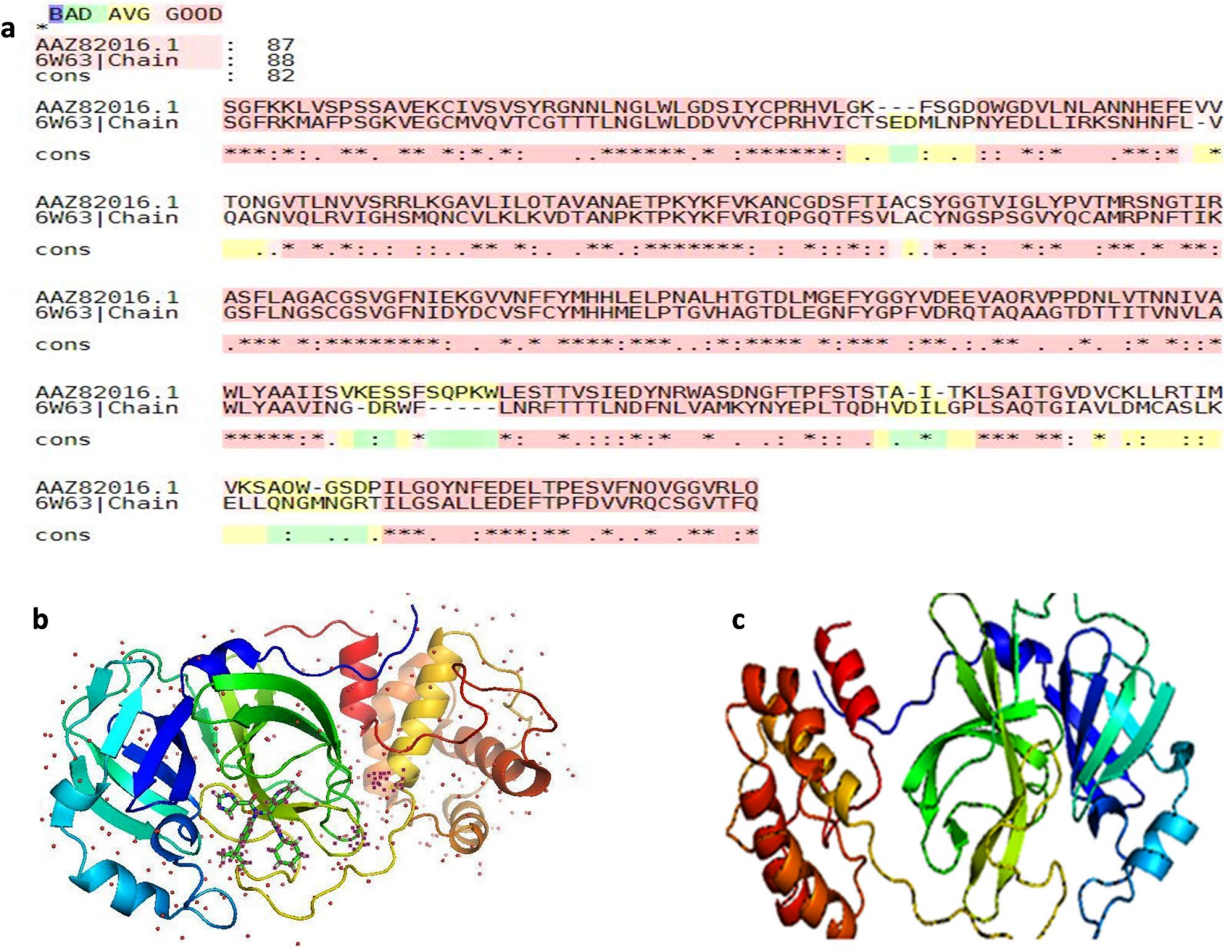
Sequence alignments between 3CLpro of SARS-CoV-2 and avian infectious bronchitis virus: (**a**) result of M-Coffee server, (**b**) 3D structure of SARS-CoV-2 3CLpro, and (**c**) 3D structure of avian infectious bronchitis virus 3CLpro.

In the first step, sequence alignment was carried out between the 3CL protease of the avian infectious bronchitis virus of the genus *Gammacoronavirus* (used in the training set of machine learning models) and the 3CLpro of SARS -CoV-2 using M-Coffee server. The results of M-Coffee depend on consistency considering incorrect alignments are less likely to be consistent than correct ones. Alignment accuracy was calculated with Column Score (CS) using the aln compare program. CS is the proportion of columns of residues correctly aligned between the test and reference alignments^15^. The score was 825 for the two given sequences and both sequences showed an excellent similarity which predicts that the protein used for the predictive model and another protein for molecular docking were similar (Fig. 2). Both proteins share similar kinds of physicochemical parameters as evaluated by the ProtParam tool of ExPASy (Table 1).

**Table 1:** Physicochemical parameters of SARS-CoV-2 3CLpro and avian 3CLpro of avian infectious bronchitis virus.

Parameters	SARS-CoV-2 3CLpro	Avian 3CLpro (Infectious bronchitis virus)
Mol. Weight	33,462.96	39,229.60
No. of amino acids	307	355
Theoretical pI	5.95	6.35
Instability index (II)	27.65	41.98
No. of Negatively Charged Residues (Asp + Glu)	26	31
No. of Positively Charged Residues (Arg + Lys)	22	29
Aliphatic Index	82.12	88.93
Grand average of Hydropathicity (GRAVY)	− 0.019	0.007
Atomic Composition	Carbon-1499; Hydrogen-2318; Nitrogen-402; Oxygen-445; Sulfur-22	Carbon-1502; Hydrogen-2323; Nitrogen-397; Oxygen-450; Sulfur-10
Amino Acid Composition	Ala-17 (5.6%); Arg-11 (3.6%); Asn-21(6.9%); Asp-17(5.6%); Cys-12 (3.9%); Gln-14 (4.6%); Glu-9 (2.9%); Gly-26; (8.5%); His-7 (2.3%); Ile-11 (3.6%); Leu-29 (9.5%); Lys-11 (3.6%); Met-10 (3.3%); Phe-17 (5.6%); Pro-13 (4.2%); Ser-16 (5.2%); Thr-24 (7.8%); Trp-3 (1.0%); Tyr 11(3.6%); Val-27 (8.8%)	Ala-25 (7%); Arg-14 (3.9%); Asn-23 (6.5%); Asp-12 (3.4%); Cys-9 (2.5%); Gln-9 (2.5%); Glu-19 (5.4%); Gly-28 (7.9%); His-6 (1.7%); Ile-22 (6.2%); Leu-28 (7.9%); Lys-15 (4.2%); Met- 4 (1.1%); Phe-16 (4.5%); Pro-13 (3.7%); Ser-31(8.7%); Thr-24 (6.8%); Trp-6 (1.7%); Tyr-11 (3.1%); Val-33 (9.3%); Pyl-3 (0.8%); Sec-2 (0.6%)

**Figure 3:**
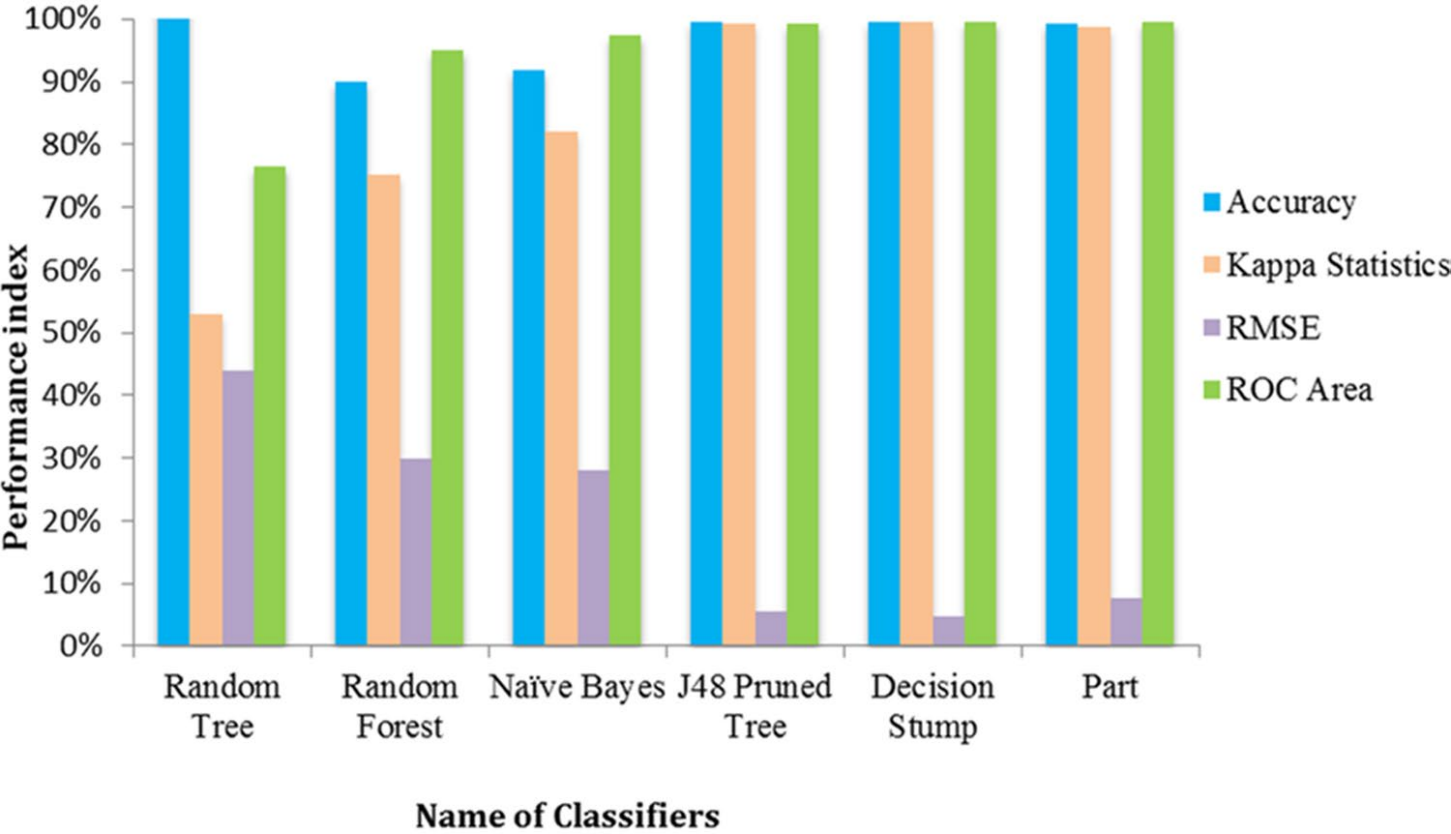
Predictive performance evaluation of classifiers using machine learning.

For the construction of the machine learning model, selected training set against 3CLpro of SARS coronavirus was evaluated by applying six different classification algorithms^16,17^, viz., Decision Stump, Random Forest, Random Tree, J48 Pruned Tree, Naive Bayes, and Part. Decision Stump classifier was selected based on the results of auto ML and the rest classifies were evaluated as they were reported frequently in the drug discovery process. Out of 1395 compounds, the number of correctly classified instances (TP + TN) of the Decision stump classifier was 1392, whereas 1391were for J48 Pruned Tree, 1387 for Part, 1257 for Random Forest, 1284 for Naive Bayes and 1125 for Random Tree (Table 2). Based on the performance index, for all applied classifiers summarized in Fig. 3, Decision Stump classifier was further selected for screening. The value of the Kappa statistics measure the consistency between the actual and the model classes, and the value 1 suggests an absolute agreement between the “ground truth” and classifier models’ classification. Model by the Decision Stump showed the highest Kappa statistic value of 0.9948 and last of RMSE value of 0.0463; followed by J48 Pruned Tree classifier having a Kappa 0.993 and last of RMSE value 0.0535 Random Tree with the lowest Kappa statistic value 0.5289 and the highest RMSE value 0.4399. The ROC curve shows the performance of a binary classifier model as its discrimination threshold is varied. The ROC curve of the present model was initially very close to the true positive rate axis which reflects minimizing false positive rate (maximizing specificity) and maximizing true positive rates (maximizing sensitivity). The highest ROC values for Decision Stump were 0.996 followed by Part 0.996, J48 Pruned Tree 0.992, Naïve Bayes 0.973, Random Forest 0.951, and lowest ROC values for Random Tree 0.764. The AUC represents the probability that the active class predicted by the classifier models for a randomly selected compound will exceed that of a randomly selected non-active class. The area under the ROC curve will be 1 if there is no overlapping among the distributed classes. Out of 1528 antiviral compounds, 356 compounds were predicted as active by the model and considered for further study.

**Table 2:** Performance comparison of classifiers and their predictive accuracy.

Classifier name	Accuracy (%)	Correctly classified instances	Incorrectly classified instances	True positives (TP)	True negatives (TN)	False positives (FP)	False negatives (FN)
Decision stump	99.78	1392	3	402	990	3	0
Random forest	90.11	1257	138	311	946	94	44
Random tree	80.65	1125	270	268	857	137	133
J48 pruned tree	99.71	1391	4	401	990	4	0
Naïve bayes	92.04	1284	111	399	885	6	105
Part	99.43	1387	8	401	986	4	4

Drug-like properties of compounds were calculated by analyzing its structural properties and applying certain drug-likeness rules. To get a potent drug-like compound, further screening was done by applying 5 drug-like rule filters viz., Lipinski's, Veber, Ghose, CMC-50, and BBB filter. According to Lipinski's "drug-like", molecules should have Log P ≤ 5, molecular weight ≤ 500, number of hydrogen bond acceptors ≤ 10, and number of hydrogen bond donors' ≤ 5. Veber rule predicts the oral bioavailability of potential drug candidates and it states a compound should have rotatable bonds < 12 and polar surface area < 140. Ghose includes the criteria like partition coefficient log P in − 0.4 to + 5.6 ranges, molar refractivity from 40 to 130, molecular weight from 180 to 480, and the number of atoms from 20 to 70^18^. CMC-50 predicts the drug-like indices of a molecule by comparing them with the Comprehensive Medicinal Chemistry (CMC) database and states that a compound should have logP (ALOGP) between 1.3 and 4.1, molar refractivity (AMR) between 70 and 110, molecular weight (MW) between 230 and 390, and the number of atoms (nAT) between 30 and 55^19^. BBB filter predicts the permeability of the Blood–Brain Barrier. 84 efficient drug-like compounds that were screened by all the above filters were considered further screening (Supplementary Table S1).

### Molecular docking analysis and deep learning screening

**Figure 4:**
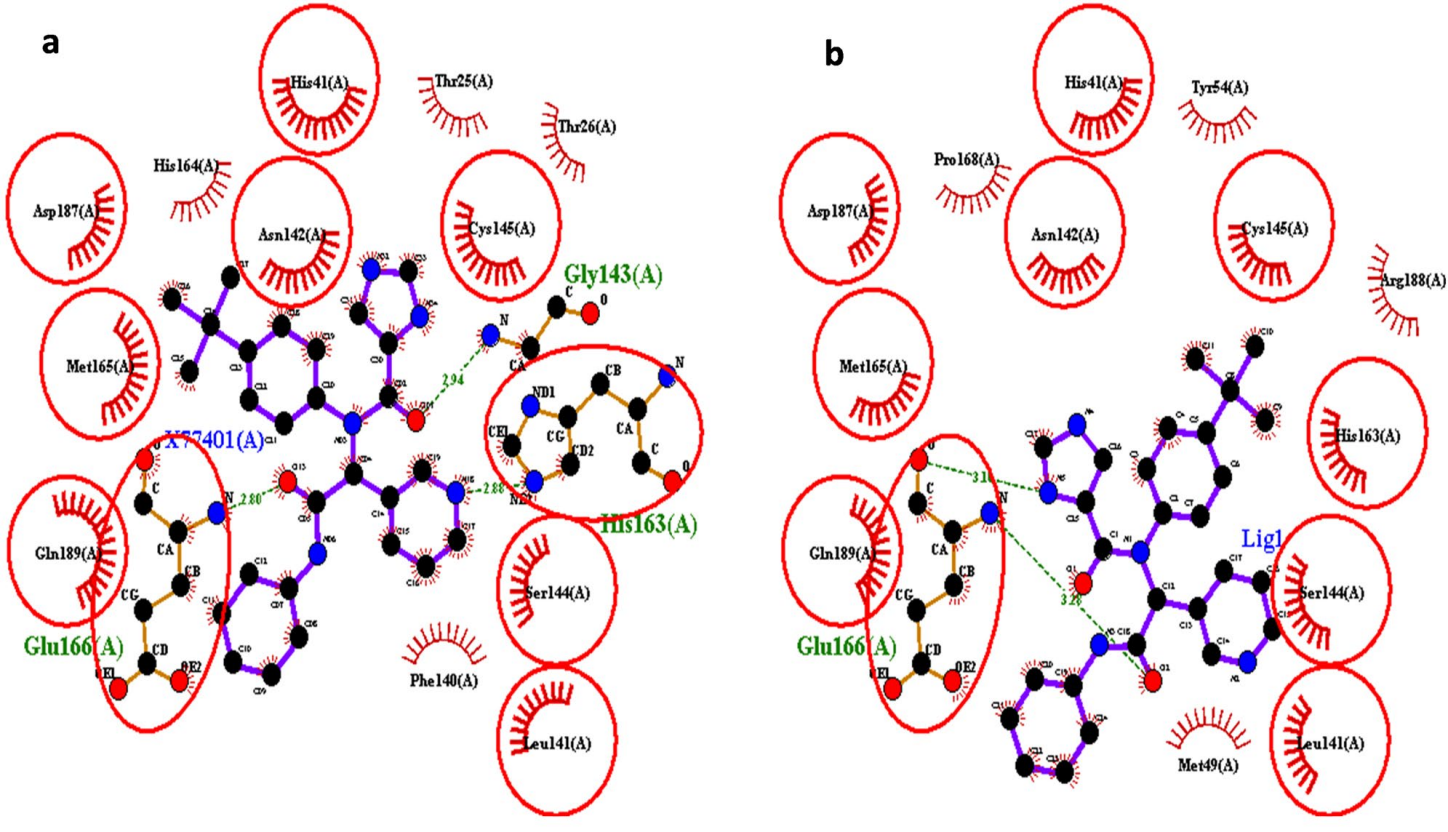
Superimposition of the active site of 3CLpro protein for docking protocol validation: (**a**) 3CLpro protein with attached reference ligand, and (**b**) protein with docked with reference ligand.

To conduct a molecular docking, a grid of 25 Å was generated over the co-crystallized peptide-like inhibitor (X77) and small-molecule inhibitor (PDB ID: 6W63). Re-docking of the co-crystallized compounds was performed to validate the docking protocols (Fig. 4). The docked complexes were superimposed to the original crystal structure to calculate the root mean square deviation (RMSD). The re-docking of peptide-like structure and small-molecule inhibitor reproduce the original pose with 1.23 Å and 0.75 Å RMSD, respectively. Lower RMSD indicates that our docking methodology is adequate and can be utilized to search small molecule inhibitors. A potential noncovalent inhibitor of SARS-CoV-2 named X77 was docked with binding pocket of main protease 3CLpro with ligand binding pose coordinates X; − 19.9080, Y; 21.1240, Z; − 29.2950. The inhibitors strongly bind to the main protease through Glu 166, Gly 143, and His 163 a.a with − 7.4 kcal/mol binding affinity. Out of 84 screened compounds, 41 showed high binding affinity and closer interactions with the conserved catalytic dyad residues (Glu 166(A), Gly 143(A), and His 163(A) as compared to reference ligand (Supplementary Table S2).

**Figure 5:**
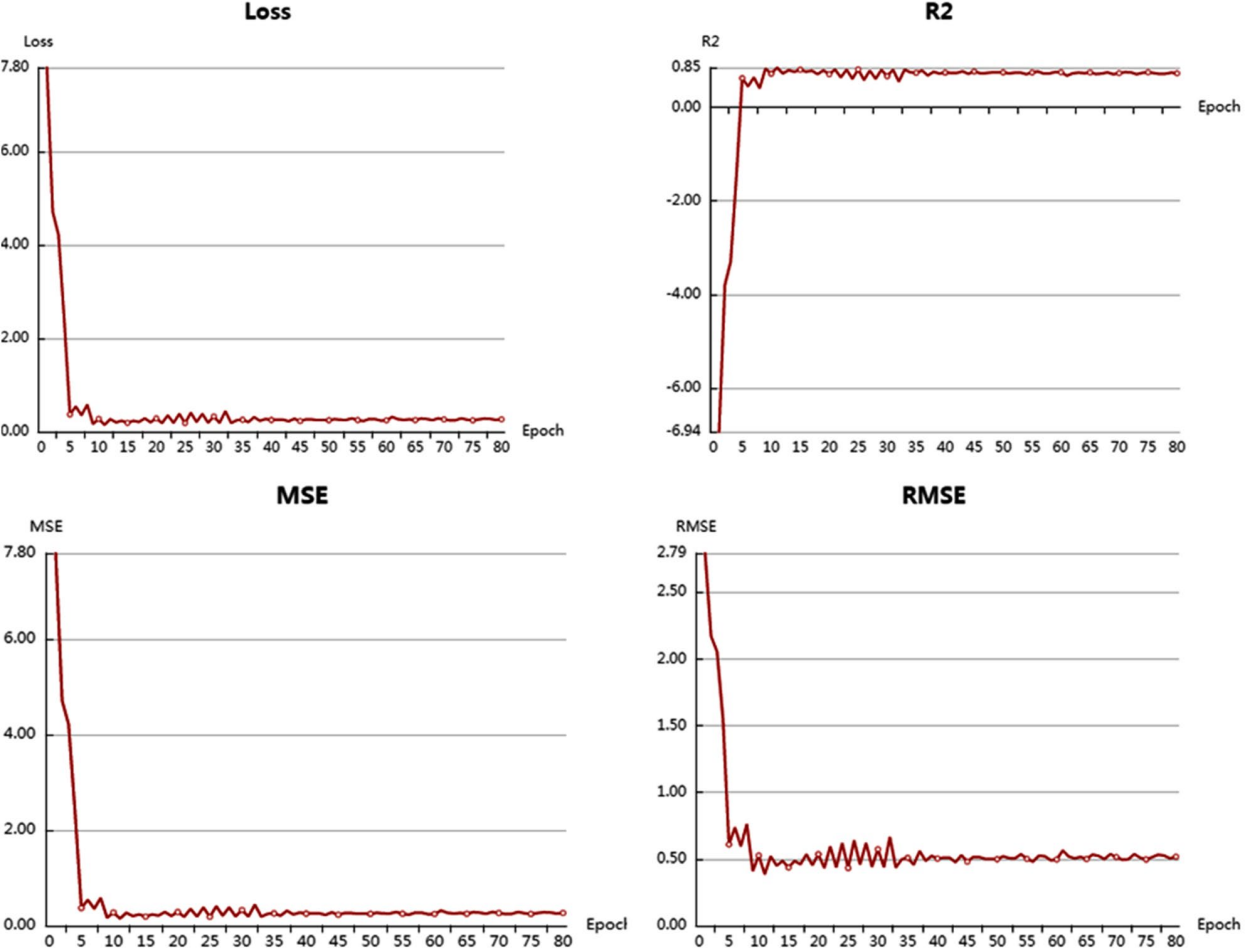
Statistical parameters of the deep learning screening model.

Further, a deep learning model was constructed to screen the compounds to get compounds with better IC_50_ value than the reference inhibitor used in molecular docking. The model was evaluated with a range of statistical parameters and good performance was observed with R2 value (0.85), MSE (7.80), and RMSE (2.79) (Fig. 5). 22 compounds were screened by the model having a score higher than the reference molecule (4.29) (Supplementary Table S3).

### SAR and functional group analysis

**Figure 6:**
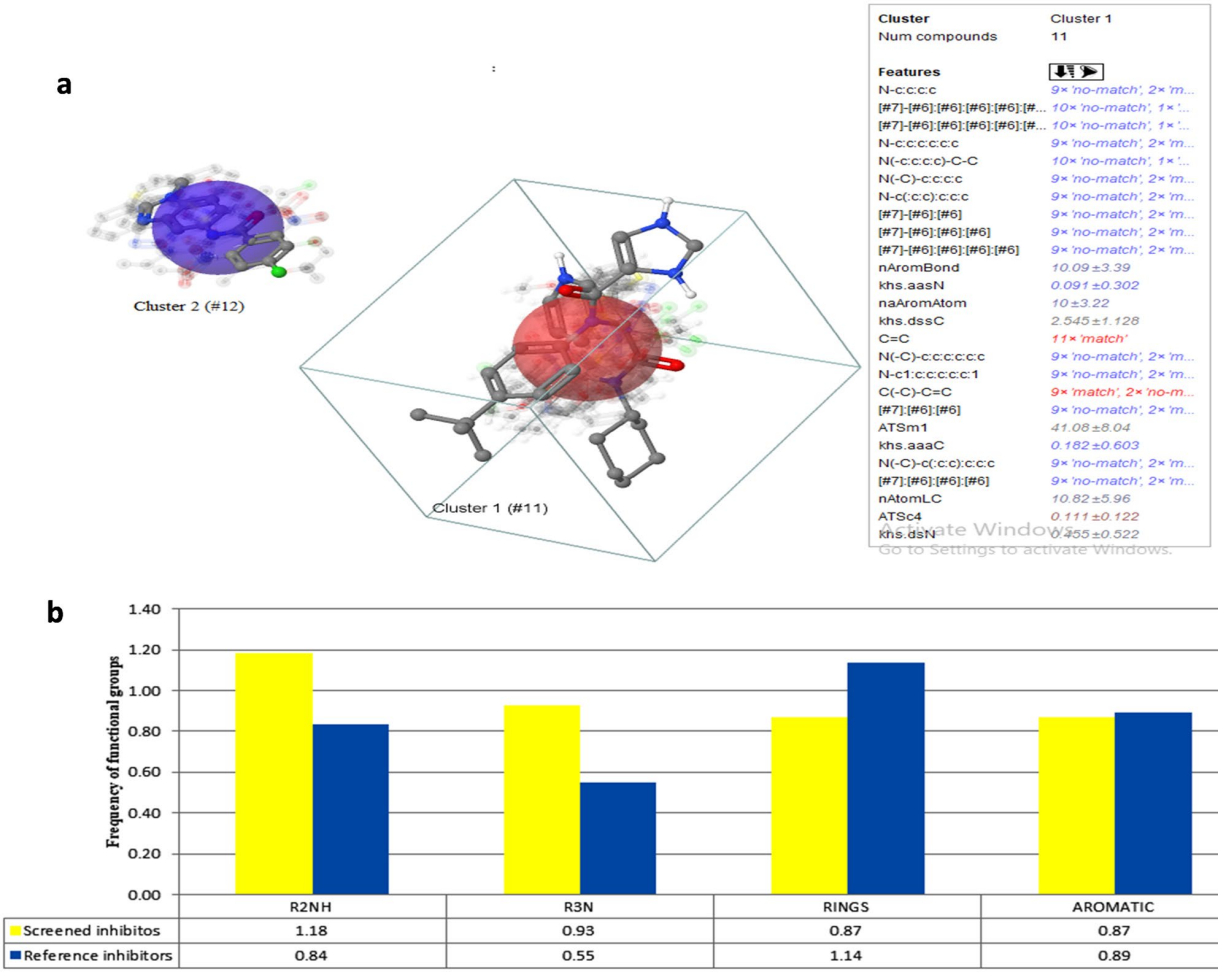
SAR and Functional group analysis: (**a**) cluster analysis in CheS-Mapper software, and (**b**) frequency of functional groups in screened inhibitors of cluster 2 and established reference inhibitors of 3CLpro.

Thereafter, screened 22 compounds were further analyzed (considering their maximum sub-structural fragments) by chemical space mapping in CheS-Mapper software. All the compounds were analyzed by considering 413 chemical properties. Along with the above 22 compounds, 12 established inhibitors of 3CLpro obtained from PDB (Supplementary Table S4) were also analyzed for their common structural fragments. The 34 compounds were mapped in two distinct clusters -16 in cluster 1 and 18 in cluster 2. As such no common fragment was found in cluster 1, compounds in cluster 2 (5 compounds were established inhibitors and 13 compounds were screened by the current study) were considered for further analyses. 61 of 413 features having equal value for each compound were not included for mapping. In cluster 2, out of 18, 5 compounds were established inhibitors and 13 compounds were screened by the current study. Thereafter, the group frequency of some vital functional groups was analyzed for both the established inhibitors and the screened compounds (Supplementary Table S5). Among them, four comparable functional groups were found to have a significant presence in reference inhibitors as well as in screened compounds (Fig. 6). The first group was R2NH (amine), followed by tertiary amines (R3N), rings, and aromatic. Amines are considered weak bases and most of the drugs have amine as functional groups as they get easily ionized in slightly acidic and alkaline pH of the gut as well as in blood. They can also easily equilibrate between the ionized and non-ionized forms. They can cross the cell membrane in non-ionized forms, whereas the ionized form allows good water solubility that ultimately promotes strong protein–ligand interaction^20^. The screened compounds contain a high frequency of amines (1.18) than the reference inhibitors (0.83). Secondary amines have N–H groups and act as a hydrogen bond donor that facilitates strong binding of the compound with the target protein^21^. The presence of secondary amine was more frequent in the screened compounds (0.92) comparing with the reference inhibitors (0.54). Aromatic rings are commonly involved in Van der Waals interactions with the binding site atoms and can also interact with an aminium (cation formed by protonation of an amine) or quaternary ammonium ion with the help of induced dipole interaction or hydrogen bonding^20^. The presence of rings at higher numbers reflects the chemical diversity of the screened compounds, whereas the presence of aromatic rings indicates their drug-like property. The screened compounds have a comparable frequency of aromatic rings (0.87) with the reference inhibitors (0.89).

**Figure 7:**
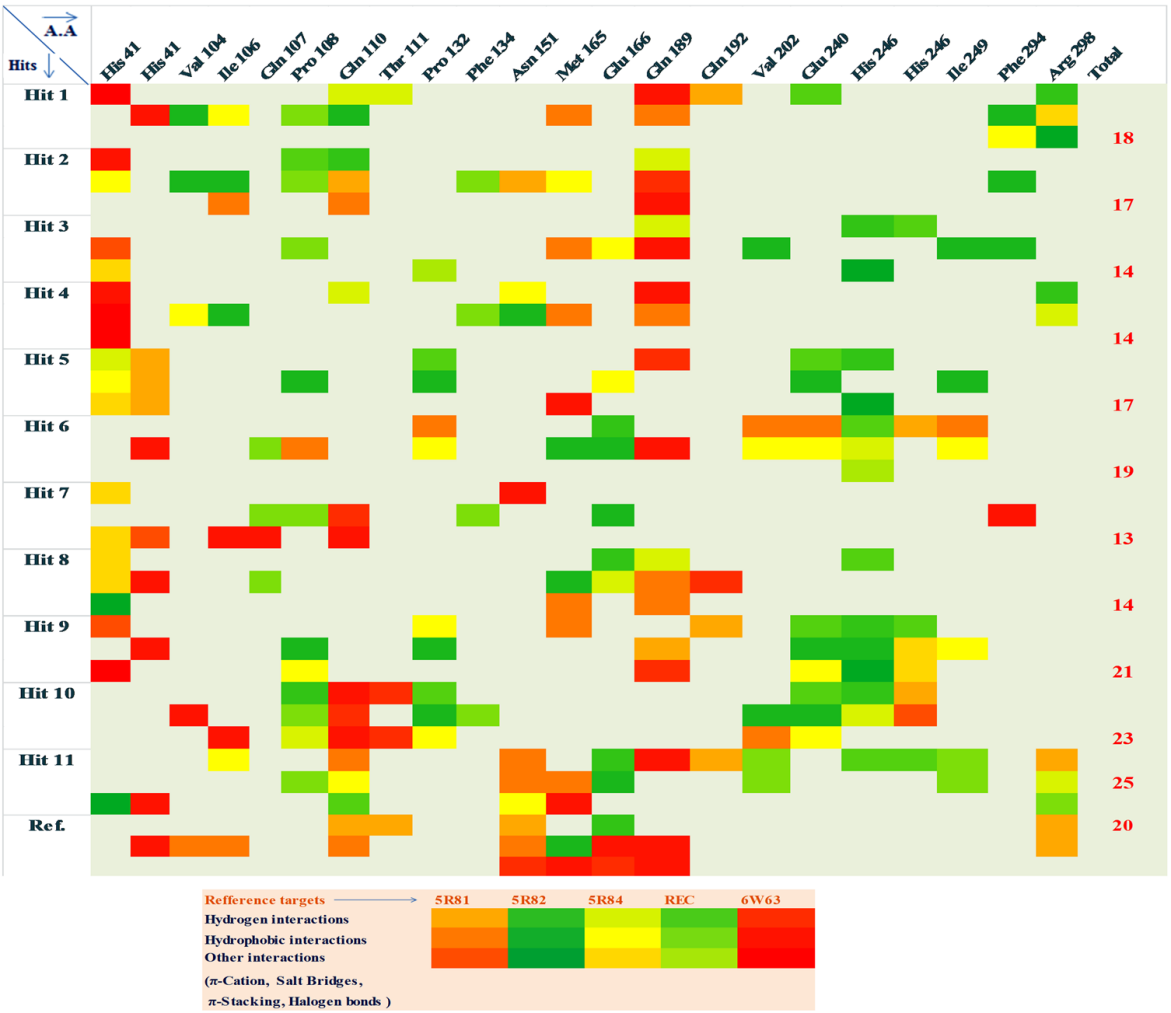
Hit map analysis depicting the interaction profile of screened compounds.

Further, docking experiment was again conducted to check the binding potential of screened compounds of cluster 2 with all other four reference PDB structures (5r81, 5r82, 5r84, and 5rec) of 3CLpro. All the screened compounds showed higher binding potential in terms of binding energy, compared with the reference ligand (Supplementary Table S6). Interaction profiles of all the docked complexes with lower RSMD values were further checked. The hit map profiles of all the interactions (hydrophobic, hydrogen, pie, halogen and base stacking (Supplementary Table S7) showed that the ligand number hit-9, hit-10, and hit-11 showed higher binding potential with 3CLpro (Fig. 7). From the above experiments, 3 out of 1528 compounds were considered as the potential hits against 3CLpro.

### Molecular dynamics simulation

**Figure 8:**
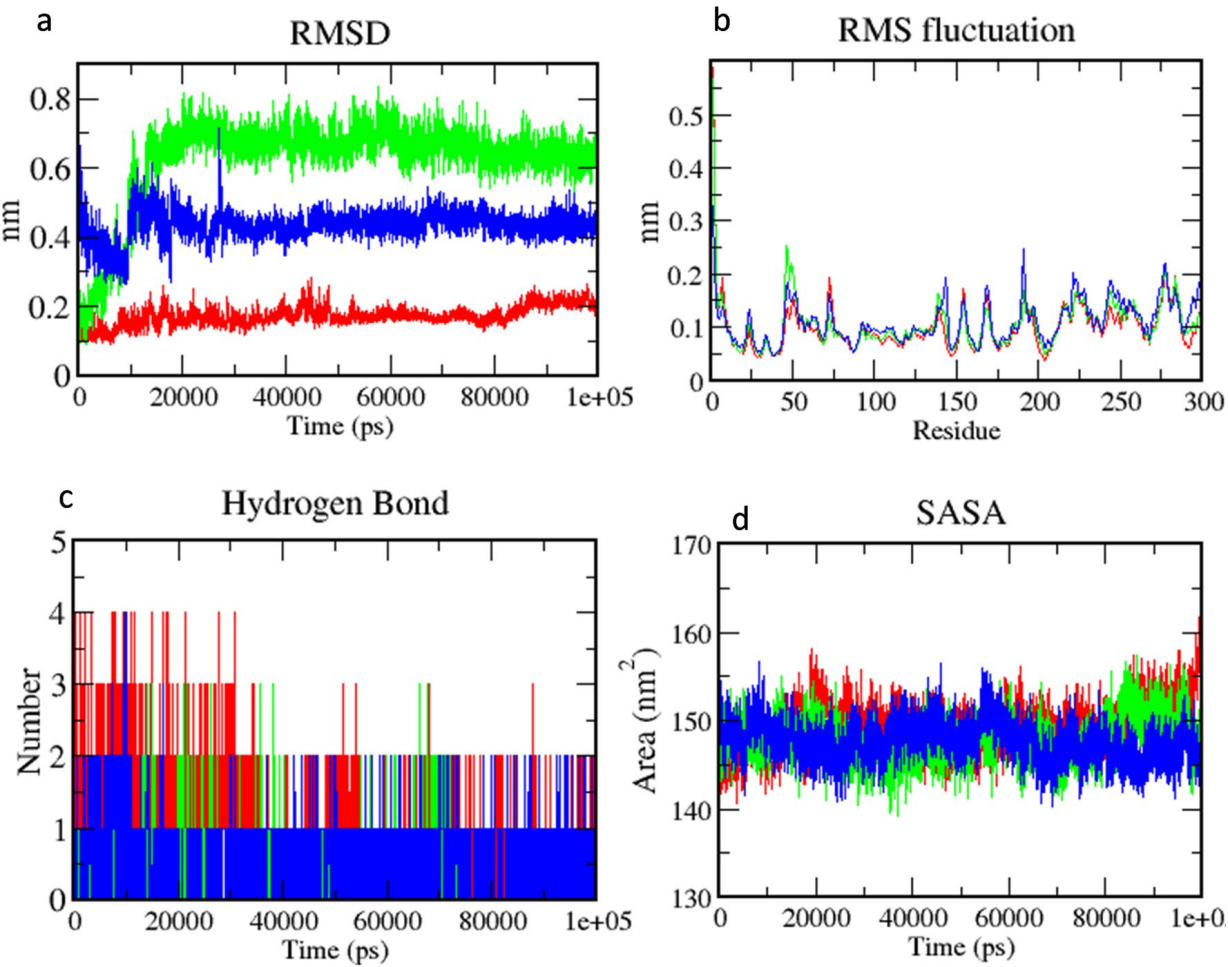
Binding stability analysis of the screened lignads during 100 ns molecular dynamics simulation (hit 9-green, hit 10—blue, reference—red): (**a**) Root Mean Square Deviation (RMSD), (**b**) Root Mean Square (RMS) Fluctuation, (**c**) hydrogen bond analysis, and (**d**) Solvent Accessible Surface Area (SASA).

In the final step, Molecular Dynamics (MD) simulations were carried out for 100 ns to analyzing the structural changes observed during the course of inhibition^22^. Analysis of Root Mean Square Deviation (RMSD) shows all complexes are stable in 100 ns simulation. The average value of RMSD is 0.62 nm (green) for hit 9, 0.43 nm (blue) for hit 10, respectively as compared to the reference 0.17 nm (red). The Root Mean Square Fluctuation (RMSF) indicates occurrence of local changes along with the protein chain residues at specific temperature and pressure. RMSF shows the fluctuation of 0.2 nm in amino acids during the 100 ns trajectory period. Hydrogen bonds play a critical role in binding a ligand to a receptor, which affects drug specificity, metabolization, and adsorption of a drug. Therefore, the total numbers of hydrogen bonds were estimated for 100 ns simulation period. Around 4 hydrogen bonds were observed in the reference while hit 9 and hit10 showed 3 and 4, respectively. Further, the average value of SASA were 147.84 ± 1.99 nm^2^ (green), 147.64 ± 1.77 nm^2^ (blue) and 149.29 ± 2.17 nm^2^ (red) for hit 9, hit 10, and reference these values show the extent of the conformational changes occurring during the interaction. The results showed the assessable surface area of screened compounds similar to the reference compound in water system which indicates the equivalent stability of hits compounds as compared to reference (Fig. 8).

## Discussion

Since 3CLpro is involved in the replication of SARS-CoV-2, inhibition of its function may be an effective strategy to control SARS-CoV-2 infection. In this regard, experimentally validated HIV protease inhibitors could be tested against SARS-CoV-2^10^ by targeting 3CLpro function. HIV protease inhibitors like Lopinavir/ritonavir are reported effective against SARS-CoV 3CLpro^14^. Since both SARS-CoV-2 and HIV belong to the positive sense RNA virus group it is logical to apply the same principle for SARS-CoV-2 also. In the genomic structure of SARS-CoV-2, ORF1a and ORF1b occupy two-thirds of the genomes at the 5′ end. ORF1a and ORF1b are translated into two polyproteins 1a and 1b (pp1a and pp1ab) through a ribosomal frame shift sequence. These polyproteins are further processed by 3CLpro and PLpro proteases to produce nonstructural proteins. Thus, blocking 3CLpro is a promising approach that will ultimately stop viral replication machinery. Some reported virtually screened compounds against SARS-CoV-2, 3CLpro are Velpatasvir, Ledipasvir, Prulifloxacin, Bictegravir, Nelfinavir, and Tegobuvi, Pimozide, Ebastine, Rupintrivir, Bepridil, Sertaconazole, Rimonabant, and Oxiconazole^23^. From current work, two potent inhibitors (CID 2301119, 948801, 859639), have been screened against 3CLpro of SARS-CoV-2 by in silico screening. Above identified inhibitors should have strong antiviral property against SARS-CoV-2, as they were screened by a comprehensive machine learning model development against 3CLpro using the dataset of avian Infectious Bronchitis Virus (IBV) (avian coronavirus). Protein sequences of 3CLpro of both viruses show on sequence similarity analysis which indicates similar molecular function. Our machine learning model had a ROC area of 0.99 which reflects high accuracy of the model in terms of both sensitivity and specificity. All the inhibitors were also screened with excellent drug-like and ADMET profiles by five drugs-like filters (Lipinski's, Veber, Ghose, CMC-50, and BBB filter). The two screened compounds also show strong binding potential with 3CLpro and share common structural clefts with the established inhibitors. They have a high frequency of antiviral functional groups like R2NH (amine), followed by tertiary amines (R3N), rings, and aromatic. During the MD simulation of 100 ns, only hit 9 and hit 10 showed high RMSD, RMSF values that reflect their stability profile during the course of inhibition. Their hydrogen bonding profile showed that they can bind very tightly with 3CLpro whereas the SASA analysis results showed that they have least exposed surface area comparing with the reference ligand which indicates their stability as complex with 3CLpro. The first screened hit compound (hit-9 in Fig. 7) is 4-{[5-(2-Nitrophenyl)-2-furyl] methylene}-3-phenyl-5(4H)-isoxazolone (CID-230119). We found a total of 73 PubChem bioassays about this compound showing activity against viruses Dengue virus-2 16,681-PDK53 and Macacamulatta polyomavirus1^24^. Previously, Phenyl oxazole compounds have been reported against 3CLpro as these aromatic moieties can bind to S1 or S2 site of SARS protease by forming H-bonds and hydrophobic interactions. The compound has also binding potential with His-41, of His41-Cys145 dyad of 3CLpro which is critical for inhibiting the catalytic activity of the enzyme. The second screened hit compound (hit-10) is 4-Chloro-N-(1-methyl-1H-benzimidazole-5-yl) benzamide (CID-948801). It has been reported as an active compound in 33 PubChem bioassays^25^. The compound was found to have hydrogen bonds with Arg298, a critical residue for maintaining the dimer structure of the enzyme and also regulates the catalytic activity. All the screened compounds have been reported as active against 3C-like protease of IBV, which reflects the strong possibilities they can be potential hits inhibitors against 3C-like protease of SARS-CoV-2. Therefore, the two newly identified inhibitors could serve as promising drug candidates against SARS-CoV-2.

## Methods

### Sequence similarity analysis

Sequence alignment analysis was done to evaluate the similarity between the 3CL protease of avian infectious bronchitis virus (IBV) that has been used in the training set of machine learning models and the 3CLpro of SARS CoV-2 that will be used for the molecular docking process. M-Coffee extension of T-Coffee multiple sequence alignments (MSA) server was used for this purpose. This server outperforms on reference datasets: HOMSTRAD, Prefab, and Balibase. Physicochemical parameters of proteins including isoelectric point, instability index, grand average of hydropathicity (GRAVY), amino acids, and atomic compositions were investigated using the ProtParam tool of ExPASy^15^.

### Dataset

In the present work, the compounds from a QFRET-based primary biochemical high throughput screening assay (AID-1706) against 3CLPro of SARS coronavirus^16^ are used as a training set. As large number of datasets is not available against 3CLpro of SARS CoV-2, the present dataset of avian infectious bronchitis virus (IBV) 3CLpro is selected for construction of the machine learning model. In this bioassay, the sum of two values was considered as cut-off parameters for classifying the compounds, viz., (i) the average percent inhibition of all compounds tested, and (ii) three times their standard deviation. In PubChem bioassays, activity score is assigned as a normalized score between 0 and 100 where the most active result rows have scores closer to 100 and inactive closer to 0. In the present bioassay, activity score between 0 and 15 were classified as inactive and between 25 and 100 as active. 405 active compounds and 1000 inactive compounds were considered for generating the QSAR model. Further, 1528 anti-HIV1 compounds were selected for screening as a test set from NCI AID antiviral assay (AID-179)^26^. From this bioassay, compounds that provide at least 50% protection by inhibiting the HIV1 infected human CEM cells were considered.

### Descriptor calculation and dataset pre-processing

The entire compound collection of training and the test set was converted into a 3D-standard data format (3D-SDF) to simplify the molecular-input line-entry system (SMILES) format by using O’babel software^27^. Further, all compounds were introduced to chemical descriptor calculation using PaDel software. Initially, a total of 2382 descriptors were extracted, which included 1D, 2D, and fingerprint features. The entire dataset was processed before building the model in WEKA software^28^. Several functions in WEKA like Correlation, Attribute, Eval, and Replace Missing Values were considered for the feature selection process. Feature selection is necessary to make a better performing predictive model.

### Predictive model and model evaluation

A machine learning model was developed using the training set compounds applying six different classifiers with a tenfold cross-validation approach. Combinations of manual and automatic methods were applied for the selection of appropriate classifier. 5 ML classifiers that are normally used for drug development were applied on the training set, viz., Random Forest, Random Tree, J48 Pruned Tree, Naive Bayes, and Part. Random forest classifier acts as tree predictors, where every single tree differs on the values of a random vector and with the identical distribution for each tree in the forest. J48 algorithm built a C4.5 decision tree and each time when it executes, an instance of the class is created by allocating memory for constructing and storing the decision tree classifier. PART classifier generally decreases the class of existing classified data to just a single class, and pick up the data using any information from other classes. Random Tree is a supervised classifier that shots the input feature vector and classifies it with each tree in the forest^29^. Finally, it outputs the class label that has obtained the majority of “votes”. Auto Machine Learning (AutoML) method was also applied that allows automatic selection of the most accurate and comprehensible ML algorithm during the model development^30^. The decision stump algorithm was selected by the AutoML function. The one-level decision tree was made by the decision stump algorithm in which the root level split based on a specific attribute. Model performance of every model was assessed by accuracy statistics and Receiver Operating Characteristic (ROC graph) which is a graphical plot of True Positive Rates (TPR) vs. False Positive Rates (FPR) for a binary classification system. Confusion matrix and evaluation statistics, including accuracy of the prediction, Kappa statistic and mean absolute error were further checked. Accuracy of the model was evaluated by using the following equations:

$$\begin{aligned} {\text{Accuracy }} & = \frac{{{\text{TP}} + {\text{TN}}}}{{\left( {{\text{TP}} + {\text{TN}} + {\text{FP}} + {\text{FN}}} \right)}} \\ {\text{Sensitivity}} & = \frac{{{\text{TP}}}}{{\left( {{\text{TP}} + {\text{FN}}} \right)}} \\ {\text{Specificity}} & = \frac{{\left( {{\text{TN}}} \right)}}{{{\text{TN}} + {\text{FP}}}} \\ {\text{MCC}} & = \frac{{\left( {\text{TP*TN}} \right) - \left( {\text{FP*FN}} \right)}}{{\sqrt {\left( {{\text{TP}} + {\text{FP}}} \right)\left( {{\text{TP}} + {\text{FN}}} \right)\left( {{\text{TN}} + {\text{FP}}} \right)\left( {{\text{TN}} + {\text{FN}}} \right)} }} \\ \end{aligned}$$

TP = True Positives; FP = False Positives; TN = True Negatives; FN = False Negatives.

The Area under Curve (AUC) value was also evaluated which denotes the probability that a classifier will rank a randomly chosen active compound higher than a randomly chosen inactive compound^31^.

### Dug-likeness and ADMET screening

Further, all the screened compounds were filtered by evaluating their Drug-like and ADMET properties using the DruLiTo software^32^. Five filters were selected for this purpose, viz., Lipinski's, Veber, Ghose, CMC-50, and BBB filter.

### Molecular docking

The 3D structure of SARS-CoV-2 main protease was retrieved from Brookhaven Protein Data Bank with PDB ID-6W63. The receptor was bound with native ligand X77, a non-covalent inhibitor. The grid parameters of native ligand were calculated by using Chimera 1.8. PyRx graphic user interface (GUI) version 0.8.was used for further screening by docking using AutoDockVina program^33^. Root Mean Square Deviation (RMSD) values were calculated to compare the accuracy of the predictions of the experimental structure. The grid parameters used for docking analysis were as under:

- Center coordinates: X = − 51.12, Y = − 6.04, Z = − 19.76.
- Dimensions (Å): X = 9.15; Y = 20.55; Z = 35.04.

### Screening by deep learning model

Additional screening was done by a deep learning predictive model using deep learning online server^34^. The dataset CHEMBL3927, having SARS coronavirus 3C-like proteinase inhibitors with IC_50_ values was used as a training set^35,36^. Regression analysis was considered for building the model and its efficacy was measured in terms of R squared (R^2^), Mean squared error (MSE), Root MSE (RMSE), and Mean absolute error (MAE).

### Structural activity relationship (SAR) mapping and functional group analysis

The final screening was carried out by SAR mapping using CheS-Mapper software protocols^37^. Here, all the screened compounds along with the reference ligand were mapped by clustering and multi-dimensional scaling through chemical space mapping. The Cascade K-Means algorithm of WEKA was used as a classifier, and feature selection was done by Principal Component Analysis. Finally, the maximum sub-structural fragment aligner algorithm was used for final alignment. The functional group's frequencies of all compounds were analyzed by R (version 3.4.3) under the library of “ChemmineR”^38^. Further, another docking experiment was also conducted to check the binding potential of the screened compounds with 4 different PDB structures of 3 CLpro, namely, 5r81, 5r82, 5r84, and 5rec and their interaction profile was checked by Lig profiler.

### Molecular dynamics (MD) simulation

The best-scored poses of screened complexes were further analyzed for binding stability by conducting MD simulations for 100 ns trajectory period at a constant temperature of 300 K using the GROMACS 18.7 software package. All the MD simulations were fabricated as an orthorhombic grid box (10 Å × 10 Å × 10 Å buffer) followed by the addition of Transferable Intermolecular Potential 3 Point (TIP3P) water molecules. Systems were neutralized by the addition of 4 NA + ions using the steepest descent algorithm with the Verlet cut-off scheme with maximum force 10 kJ/mol. Further, the equilibration step was carried out on NVT (constant volume) as well as NPT (constant pressure) for 100 ps time. Further, MD simulation was conducted for a 100 ns time at a constant temperature of 300 K and a constant pressure of 1 atm with a time step of 2 fs using the Parrinello-Rahman method. The generated trajectories were used to analyze each complex's behavior to access the stability of the system in the explicit water environment. The deviations of the complex system were analyzed by calculating Root Mean Square Deviation (RMSD), Root Mean Square Fluctuation (RMSF), hydrogen bonds, Solvent Accessible Surface Area (SASA)^39^.

## Supplementary information

Supplementary Information.

## Data Availability

Initial X-ray structures are available at Protein Data Bank (https://www.rcsb.org/). Datsets used from different bioassays are available at PubChem (https://pubchem.ncbi.nlm.nih.gov/). All other data are available either in the main text or as supplementary materials.
