# Supplementary material for: Virtual screening of anti-HIV1 compounds against SARS-CoV-2: machine learning modeling, chemoinformatics and molecular dynamics simulation based analysis
Source: Sci Rep. 2020 Nov 23;10:20397. doi: 10.1038/s41598-020-77524-x (PMC7683650; doi:10.1038/s41598-020-77524-x)
Supplement: Supplementary file 1 — Supplementary Information. [file 41598_2020_77524_MOESM1_ESM.pdf]

Virtual Screening of Anti-HIV1 Compounds against SARS-CoV-2: Machine Learning Modeling, Chemoinformatics and Molecular Dynamics Simulation Based Analysis

Mahesha Nand[1+], Priyanka Maiti[2+*], Tushar Joshi[3], Subhash Chandra[4*], Veena Pande[5], Jagdish Chandra Kuniyal[6], MuthannanAndavar Ramakrishnan[7*]

Supplementary Table S1 Drug -like properties of compounds screened by DruLiTo

Pubchem Ids	MW	logp	Alogp	HBA	HBD	TPSA	AMR	nRB	nAtom	nAcidic Group	RC	nRigidB	nArom Ring	nHB	SAlerts
73157	456.18	5.123	2.367	8	2	119.36	119.51	10	61	1	3	25	2	10	4
215090	196.1	2.021	0.69	2	0	15.6	67.63	0	27	0	3	17	2	2	0
**2301119**	**325.13**	**1.896**	**1.84**	**5**	**1**	**100.63**	**103.17**	**2**	**40**	**0**	**3**	**25**	**2**	**6**	**1**
638860	247.07	2.25	1.613	3	1	77.62	74.82	4	30	0	2	14	2	4	2
652480	446.18	4.614	2.908	6	1	91.59	138.86	9	58	0	4	26	4	7	2
657485	333.12	1.722	1.489	7	1	90.93	88.77	8	43	0	2	17	1	8	3
661510	352.19	1.834	0.716	6	0	57.5	108.92	8	50	0	3	20	3	6	1
675749	292.02	2.139	1.73	3	0	32.67	76.32	2	30	0	2	16	2	3	0
677475	271.05	1.037	1.53	4	0	45.03	81.19	3	29	0	3	18	3	4	1
708625	284.06	1.139	1.728	4	1	75.99	87.43	4	32	0	3	18	3	5	0
728528	270.05	1.73	0.753	5	0	61.83	76.33	3	30	0	3	19	2	5	2
736374	244.05	2.241	0.653	4	1	53.98	75.14	1	26	0	3	18	3	5	1
738894	327.05	1.7	2.118	4	2	122.31	94.4	5	35	0	3	19	2	6	1
741948	250.11	1.978	1.941	3	0	32.67	82.9	2	33	0	3	19	3	3	0
765284	279.13	2.159	0.662	3	1	38.33	92.16	3	38	0	3	20	2	4	1
778154	348.1	3.125	2.324	5	2	91.15	106.9	5	41	0	4	23	4	7	0
805223	283.08	2.144	0.081	5	0	55.84	84.52	4	34	0	3	19	3	5	2
807412	284.07	2.194	0.418	5	0	61.83	81.17	4	33	0	3	19	2	5	2

859639	321.06	2.684	1.955	3	1	71.47	90.37	6	37	0	2	16	2	4	2
880528	254.14	3.668	0.296	3	1	32.34	78.74	3	37	0	3	18	2	4	1
**948801**	**611.27**	**3.391**	**1.985**	**11**	**4**	**147.22**	**178.8**	**18**	**82**	**0**	**4**	**30**	**4**	**15**	**2**
970829	348.05	1.532	1.962	4	1	98.54	90.71	7	37	0	2	18	2	5	4
972394	278.14	-0.22	0.508	4	1	78.72	75.85	5	38	0	2	16	1	5	2
1008368	356.08	1.823	0.652	6	0	85.66	104.44	4	41	0	4	24	3	6	1
1160939	357.01	2.417	3.273	4	0	120.93	107.87	3	34	0	4	23	3	4	1
1214257	372.01	1.458	0.745	5	1	74.86	99.03	5	37	0	2	19	2	6	0
1304219	420.11	1.9	2.942	6	3	119.75	128.57	9	50	1	3	23	3	9	1
1327906	390.1	3.184	2.94	5	3	110.52	122.25	8	46	1	3	22	3	8	1
1475477	494.04	3.652	1.922	5	2	92.73	123.16	11	50	0	2	21	2	7	1
1537038	251.07	2.034	0.843	3	0	29.54	73.5	4	31	0	2	14	2	3	2
2017227	360.04	2.014	0.869	4	0	40.62	103.44	5	38	0	3	21	3	4	1
2057118	263.09	2.265	1.55	3	0	37.38	86.62	3	33	0	3	19	2	3	1
2078229	334.13	1.748	0.639	5	0	49.85	105.07	3	43	0	4	25	2	5	1
2104090	325.1	2.904	1.926	4	1	44.7	100.31	5	39	0	3	20	3	5	1
2108058	453.1	1.751	0.563	9	2	115.86	128.66	6	51	0	5	30	4	11	0
2134372	335.04	2.27	2.322	4	1	71.62	93.37	5	36	0	2	18	2	5	0
2282752	265.11	2.216	0.856	3	0	37.38	84.08	4	35	0	3	18	2	3	1
2310813	252.09	2.237	0.737	4	0	41.9	80.71	3	31	0	3	18	3	4	2
2333509	318.11	4.409	3.37	2	1	37.3	104.43	4	39	1	3	22	3	3	3
2348921	380.03	3.122	1.324	5	0	64.45	103.31	3	38	0	4	24	3	5	0
2384236	301.12	3.433	2.858	2	0	20.31	95.42	8	41	0	2	14	2	2	1
2396278	415.06	1.52	2.392	5	1	83.06	121.04	7	46	1	3	23	3	6	0
2440506	274.11	2.357	1.108	3	1	45.05	92.38	3	35	0	3	20	3	4	1
2530053	280.02	0.97	0.617	3	1	41.46	77.87	4	28	0	2	15	2	4	0

2739954	427.01	2.449	1.182	4	1	63.78	92.54	4	36	0	3	26	3	5	3
2814665	327.11	1.946	0.121	6	1	73.86	92.46	5	41	0	3	21	2	7	2
2829992	443.04	4.558	4.961	4	3	85.25	122.36	8	47	0	2	20	2	7	3
2890716	307.12	3.391	2.051	2	1	29.1	85.11	6	38	0	2	17	2	3	1
2898168	269.12	1.587	2.041	2	1	29.1	83.26	1	36	0	3	21	2	3	1
2898209	400.1	2.572	2.54	4	2	66.58	121.85	7	48	0	3	22	3	6	0
2917531	265.12	1.081	2.026	4	2	68.62	80.15	4	37	0	2	15	1	6	1
2999977	266.11	2.629	1.183	4	0	41.9	85	3	34	0	3	19	3	4	2
3091264	464.96	4.16	4.553	5	3	94.48	120.29	10	42	0	2	18	2	8	4
3206295	499.19	0.251	0.849	10	1	105.06	147.05	12	62	0	5	29	5	11	0
3555173	383.1	1.566	0.065	6	2	74.16	110.59	5	45	0	4	25	3	8	2
3590295	496.1	1.36	1.521	8	2	117.6	141.66	8	55	0	4	29	4	10	0
3811656	364.06	2.313	0.555	5	0	76.43	102.19	6	41	0	3	20	2	5	2
3929515	380.15	2.878	0.846	4	0	49.74	123.69	3	49	0	5	30	3	4	1
4220289	319.07	2.555	1.401	5	2	131.96	85.16	7	35	0	2	16	2	7	3
4330749	369	2.654	3.008	4	1	71.62	99.06	5	36	0	2	19	2	5	0
4437603	318.06	2.858	1.999	3	0	57.97	93.19	4	36	0	3	19	2	3	2
4621376	271.12	2.088	0.725	4	0	47.89	80.12	7	37	0	2	14	2	4	2
4679126	482.11	2.047	1.216	9	1	140.03	134.89	8	56	0	4	29	3	10	4
4794522	345.08	2.376	1.927	5	1	64.63	93.03	5	38	0	3	22	2	6	3
4868361	303.08	1.151	1.193	5	2	61.44	88.13	7	35	0	2	15	2	7	2
4907806	366.12	1.593	2.144	6	1	82.51	108.2	4	44	0	4	25	4	7	0
4961646	292.06	1.655	0.072	5	0	51.13	79.64	4	33	0	3	18	2	5	2
4961648	308.04	2.02	0.672	4	0	67.2	85.81	4	33	0	3	18	2	4	2
5349620	377.1	2.251	0.145	5	0	102.11	101.83	4	43	0	5	28	2	5	4
5928454	337.13	2.432	1.297	5	0	55.84	103.3	6	44	0	3	21	2	5	3
6268409	348.12	2.85	2.976	3	1	46.53	111.31	6	43	1	3	22	3	4	3

9563955	391.09	2.878	2.075	5	2	70.56	110.23	7	45	0	2	20	2	7	2
9566932	406.03	1.957	1.54	6	1	138.72	110.78	5	41	0	3	23	2	7	4
9566958	406.03	1.957	1.54	6	1	138.72	110.78	5	41	0	3	23	2	7	4
16682428	307.09	2.602	2.598	3	2	87.53	85.19	4	38	0	2	16	2	5	4
17379494	405.14	0.096	0.602	8	1	96.56	113.79	7	51	0	3	23	2	9	0
17584476	339.11	1.34	0.211	6	0	65.07	98.46	4	42	0	4	24	2	6	1
22583081	383.13	1.368	0.418	6	0	70.59	114.1	4	48	0	4	26	3	6	0
24761147	373.1	0.188	0.358	6	1	61.88	90.2	5	45	0	3	20	1	7	0
24789267	543.31	3.785	1.358	11	4	147.22	147.98	17	80	0	2	23	2	15	2
24791295	377.22	4.363	0.944	6	2	69.09	113.39	5	55	0	4	26	3	8	0
24819986	354.1	0.713	0.604	4	1	132.61	92.74	6	40	0	3	22	3	5	5
135549448	273.09	3.448	0.822	3	0	43.87	79.55	4	32	0	2	18	2	3	6
135549741	291.14	2.197	1.166	4	2	67.48	95.71	2	39	0	3	22	2	6	3
135549849	293.1	2.417	0.045	6	2	109.58	77.25	7	36	0	1	14	1	8	5
135550923	472.14	1.091	1.437	10	2	134.78	127.11	8	57	0	4	28	2	12	0
135599408	313.09	2.945	1.848	5	1	88.35	92.02	4	37	0	3	20	2	6	2
135614387	467.15	2.751	0.637	8	2	116.68	138.01	9	58	0	3	26	3	10	2
135615831	318.05	1.714	1.507	4	1	90.91	100.83	2	33	0	4	24	4	5	2
135650685	306.15	1.54	1.321	5	1	57.06	97.65	3	41	0	3	22	2	6	2
135735436	529.86	3.239	2.412	6	2	129.51	120.5	3	39	0	3	26	2	8	1
135795461	341.04	2.034	0.393	3	1	64.2	90.55	2	35	0	3	23	2	4	4
135802830	283.11	1.118	0	2	1	55.91	0	4	37	0	2	15	1	3	4
135829849	324.15	2.28	1.145	5	2	70.92	102.93	7	44	0	2	18	2	7	3

Supplementary Table S2 Binding energy of screened ligands with 3CLpro of SARS-CoV-2

S. No	Compounds CID	Formula	Binding affinity (kcal/mol)
1	**948801**	**C16H17ClN3O**	**-7-7**
2	3929515	C25H20N2O2	-10.7
3	4907806	C19H18N4O2S	-9.7
4	**2301119**	**C21H14N2O5**	**-9.7**
5	135549741	C18H17N3O	-9.6
6	2078229	C20H18N2O3	-9.6
7	3774350	C17H20N6O3	-9.6
	3555173	C20H18ClN3O3	-9.5
8	4330749	C16H13Cl2NO3S	-9.4
9	1160939	C16H15N3OS3	-9.3
10	2134372	C16H14ClNO3S	-9.1
11	135549448	C17H14N3O	-9.1
12	2472898	C16H17F2NO2S2	-9.1
13	2348921	C16H14Cl2N4OS	-9.0
14	738894	C16H16N3OS2	-9.0
15	2898168	C17H16FNO	-8.9
16	1214257	C15H14Cl2N2O3S	-8.8
17	4794522	C18H13F2NO4	-8.8
18	1008368	C19H16N2O4S	-8.7
19	859639	C16H16ClNO2S	-8.7

20	4437603	C16H15ClN2OS	-8.6
21	135650685	C19H22N2SN3	-8.6
22	4220289	C13H13N5O3S	-8.4
23	16682428	C15H19ClN3S	-8.3
24	2104090	C17H17ClN2O3S	-8.3
25	135716604	C19H11ClF3N3O2	-8.3
26	880528	C16H18N2O	-8.0
27	1537038	C17H18BrN2O	-7-1
28	675749	C13H13BrN2O	-7.9
29	741948	C16H14N2O	-7.9
30	2890716	C17H16F3NO	-7.9
31	765284	C18H18NO2	-7.9
32	665102	C17H23N3O2S	-7.9
33	2440506	C18H14N2O	-7.5
34	Reference	C27H33N5O2	-7.4
35	2999977	12H19N3O2S3N	-7.4
36	135616984	C17H15N4O3	-7.4
37	2282752	C17H15NO2	-7.4
38	5928454	C14H17F2NO2S2	-7.3
39	135829849	C19H22N2O3	-6.7
40	4621376	C18H16F3NO	-6.5
41	135549849	C19H16N3O	-6.1

Supplementary Table S3 Scores of screened ligands by deep learning

**Ligands**	**PubChem IDs**	**Score**
Reference	Xxj (ref)	4.29
Hit-1	2898168	4.7396
Hit-2	741948	5.0882
Hit-3	2348921	5.304
Hit-4	2017227	5.3106
Hit-5	4220289	4.319
Hit-6	765284	4.6551
Hit-7	4907806	4.9522
Hit-8	1008368	4.5572
**Hit-9**	**2301119**	**4.612**
**Hit-10**	**948801**	**4.5475**
Hit-11	859639	4.6563
Hit-12	1160939	4.3816
Hit-13	2134372	4.4527
Hit-14	17584476	4.5652
Hit-15	661510	4.6767
Hit-16	2104090	4.8346
Hit-17	4437603	4.851
Hit-18	3811656	4.8784
Hit-19	2440506	4.912
Hit-20	2310813	5.0713
Hit-21	4330749	5.0767
Hit-22	3555173	5.3024

Supplementary Table S4 Details of 12 reference inhibitors used for structural cleft analysis

Drug or cocktail	Originator company	Status and mechanisms	Clinical trials (trial posting date)
**ASC09/ritonavir, lopinavir/ritonavir, with or without umifenovir**	Ascletis, AbbVie, Pharmstandard	ASC09 is an experimental HIV-1 protease inhibitor; ritonavir and lopinavir/ritonavir are approved protease inhibitors for HIV/AIDS; umifenovir is an approved entry inhibitor against influenza	At least three trials (e.g., ChiCTR2000029603, 2/6/20)
**SC09/oseltamivir, ritonavir/oseltamivir, oseltamivir**	Ascletis, Gilead, AbbVie	See above; oseltamivir is a sialidase inhibitor approved for influenza	One trial (NCT04261270, 2/7/20)
**Azvudine**	Zhengzhou GranlenPharmaTech	Experimental reverse transcriptase inhibitor drug against HIV-1/AIDS	One trial (ChiCTR2000029853, 2/15/20)
**Various combinations of baloxavirmarboxil/favipiravir and lopinavir/ritonavir**	Shionogi, Toyama Chemical	Baloxavirmarboxil is a Cap-dependent endonuclease inhibitor and favipiravir is a guanine analog RNA-dependent RNA polymerase inhibitor approved for influenza A and B; see above	Two trials (ChiCTR2000029544, 2/3/20; ChiCTR2000029548, 2/4/20)
**Various combinations of darunavir/cobicistat alone or with lopinavir/ritonavir and thymosin α1**	Janssen, Gilead	Darunavir and cobicistat are, respectively, an HIV-1 protease inhibitor and inhibitor of cytochrome P450 (CYP) 3A enzyme, approved as a combination against HIV-1/AIDS. Thymosin α1 is an immune response boosting agent	Two trials (NCT04252274, 2/5/20; ChiCTR2000029541, 2/3/20)
**Remdesivir**	Gilead	Phosphoramidate prodrug of an adenine analog used for Ebola and Marburg virus outbreaks (similar structure to approved HIV reverse transcriptase inhibitors)	Two trials (NCT04252664, 2/5/20; NCT04257656, 2/6/20)

**Chloroquine or hydroxychloroquine**	Shanghai Zhongxi Pharmaceutical, Shanghai Ziyuan Pharmaceutical, Wuhan Wuyao Pharmaceutical	Endosomal acidification fusion inhibitor	At least ten trials (e.g., ChiCTR2000029826, 2/2/20; NCT04261517, 2/14/20)
**Methylprednisolone**	Generic	Synthetic corticosteroid that binds to nuclear receptors to dampen proinflammatory cytokines	One trial (NCT04263402, 2/10/20)
**Nterferon alfa-2b alone or in combination with lopinavir/ritonavir and ribavirin**	Biogen, Merck	Interferon alfa-2b is a recombinant cytokine with antiviral properties; ribavirin is a guanine derivative; as above	Two trials (NCT04254874, 2/5/20; ChiCTR2000029308, 1/23/20)
**Camrelizumab and thymosin**	Incyte, Shanghai Hengrui Pharmaceutical	Camrelizumab is a humanized monoclonal antibody (mAb) targeting PD-1	Two trials (ChiCTR2000029806, 2/14/20; NCT04268537, 2/14/20)
**Cilizumab**	Chugai Pharmaceutical, Zhejiang Hisun Pharmaceutical, Jiangsu Qyun Bio-Pharmaceutical	Humanized mAb targeting interleukin-6	One trial (ChiCTR2000029765, 2/13/20)

Supplementary Table S5 Frequency of functional groups in screened and reference inhibitors

Ligands	MF	MW	R2NH	R3N	ROH	RCOR	RCOOR	ROR	RCN	RINGS	AROMATIC
17	C17H16FNO	269.3134	1	0	0	0	0	0	0	3	2
20	C16H14N2O	250.2952	0	1	0	0	0	0	0	3	3
21	C16H14Cl2N4OS	381.2796	0	3	0	0	0	0	0	4	3
34	C18H14Cl2N2O2	361.222	0	2	0	0	0	0	0	3	3
37	C19H17NO5	339.342	0	1	0	1	0	3	0	4	2
42	C13H13N5O3S	319.339	2	0	0	0	0	0	0	2	2
45	C18H17NO2	279.3331	0	0	0	1	0	1	0	3	2
47	C19H18N4O2S	366.4368	0	0	0	0	0	0	0	5	1
61	C18H16N2O4S	356.3956	0	1	0	0	0	3	0	4	3
64	**C20H12N2O5**	**360.3197**	**0**	**0**	**0**	**0**	**1**	**0**	**0**	**4**	**4**
65	**C15H12ClN3O**	**285.7283**	**1**	**1**	**0**	**0**	**0**	**0**	**0**	**3**	**3**
69	$C_{16}H_{16}ClNO_2S$	321.8217	1	0	0	1	0	0	0	2	2
Reference	C27H4N5O2	430.353	4	1	0	0	0	0	0	4	1
Frequency			1.182132	0.926809	0	0.438529	0.27735	1.126601	0	0.869718	0.869718
GWS	C13H18N2O	218.2948	1	0	0	0	0	0	0	2	1
Ref	C27H32N5O283	4954.407	2	1	0	0	0	0	0	4	3
RZJ	C10H14N2O2S	226.295	0	1	0	0	0	0	0	2	1

		4									
RZS	C8H9N3	147.177 2	1	0	0	0	0	0	1	1	1
T1J	C14H13N3O	239.272 5	2	0	1	0	0	0	0	3	2
Frequency			0.83666	0.54772 3	0.44721 4	0	0	0	0.44721 4	1.14017 5	0.894427

Supplementary Table S6 Binding energy of hit ligands with different PDB IDs of 3CLpro

Ligand	PubChem IDs	5R811	5R82	5R84	5REC
Hit-1	2898168	-8.8	-7.7	-8.1	-4.6
Hit-2	741948	-7.7	-7.5	-8.2	-4.3
Hit-3	2348921	-8.8	-9.1	-9.5	-5.3
Hit-4	2017227	-8.9	-8.5	-8.6	-4.8
Hit-5	4220289	-8.2	-7.8	-8.2	-4.7
Hit-6	765284	-8.1	-7.1	-8.1	-4.4
Hit-7	4907806	-9.9	-9	-10.1	-5.9
Hit-8	1008368	-9.2	-8.4	-9.2	-5.4
Hit-9	2301119	-10.1	-10.4	-11.1	-6.4
Hit-10	**948801**	**-8.2**	**-8.5**	**-8.7**	**-4.6**
Hit-11	**859639**	**-9**	**-7.8**	**-8.5**	**-4.8**
Hit-12	Reference	-6.9	-4.9	-2.6	-1.9

Supplementary Table S7 Interaction profile of screened ligands with 3CLpro

Hit 9 with 5R81

Hydrophobic Interactions

Index	Residue	AA	Distance	Ligand Atom	Protein Atom
1	165A	MET	3.46	2883	1574
2	168A	PRO	3.61	2893	1603
3	189A	GLN	3.97	2882	1791
4	189A	GLN	3.71	2896	1792

Hydrogen Bonds

Index	Residue	AA	Distance H-A	Distance D-A	Donor Angle	Protein donor?	Sidechain	Donor Atom	Acceptor Atom
1	189A	GLN	2.56	3.36	142.04	✗	✓	2897 [O3]	1794 [O2]
2	190A	THR	2.38	2.85	107.21	✗	✗	2874 [Nox]	1802 [O2]
3	190A	THR	3.2	4.09	149.12	✓	✗	1799 [Nam]	2871 [O3]
4	192A	GLN	3.21	3.93	129.39	✓	✓	1822 [Nam]	2874 [Nox]

π-Stacking

Index	Residue	AA	Distance	Angle	Offset	Type	Ligand Atoms
1	41A	HIS	4.7	76.22	0.09	T	2878, 2885, 2886, 2888, 2889, 2890

Hit 10 with 5R81

Hydrophobic Interactions

**Index**	Residue	AA	Distance	Ligand Atom	Protein Atom
1	108A	PRO	3.97	2887	1051
2	132A	PRO	3.81	2890	1267
3	200A	ILE	3.7	2891	1885
4	202A	VAL	3.24	2877	1902
5	240A	GLU	3.72	2890	2294

Hydrogen Bonds

Index	Residue	AA	Distance H-A	Distance D-A	Donor Angle	Protein donor?	Sidechain	Donor Atom	Acceptor Atom
1	108A	PRO	2.32	2.93	168.55	✖	✖	2881 [Nam]	1049 [O2]
2	246A	HIS	2.54	3.23	124.41	✔	✔	2354 [N3]	2883 [O2]

π-Stacking

Index	Residue	AA	Distance	Angle	Offset	Type	Ligand Atoms
1	246A	HIS	4.88	82.19	1.88	T	2870, 2871, 2873, 2874, 2879

Hit 11F with 5R81

**Hydrophobic Interactions**									
Index	Residue	AA	Distance	Ligand Atom		Protein Atom			
1	110A	GLN	3.64						
2	151A	ASN	3.6	2889	1063				
3	151A	ASN	3.77	2875	1431				
4	153A	ASP	3.59	2881	1431				
**HydrogenBonds**									
Index		Residue	AA	Distance H-A					
1	158A	SER	3.1	Distance D-A	Donor Angle	Protein donor?	Sidechain	Donor Atom	Acceptor Atom
2	298A	ARG	2.31	3.84	135.71			1500 [O3]	2872 [O2]
**Halogen Bonds**				2.81	108.74			2812 [Ng+]	2873 [O2]
Index		Residue	AA	Distance					
1	295A	ASP	3.69	Donor Angle	Acceptor Angle	Donor Atom	Acceptor Atom		
				150.5	139.91	2870 [Cl]	2784 [O2]		

Hit 9 with 5R82

Hydrophobic Interactions

Index	Residue	AA	Distance	Ligand Atom	Protein Atom
1	108A	PRO	3.48	2895	1050
2	108A	PRO	3.77	2896	1051
3	132A	PRO	3.46	2894	1267
4	200A	ILE	3.57	2893	1885
5	240A	GLU	3.59	2893	2294
6	249A	ILE	3.68	2889	2381

Hydrogen Bonds

Index	Residue	AA	Distance H-A	Distance D-A	Donor Angle	Protein donor?	Sidechain
1	203A	ASN	3.32	3.79	110.64	✔	✔
2	203A	ASN	2.2	2.7	111.86	✖	✔
3	246A	HIS	3.23	3.92	126.98	✔	✔

π-Stacking

Index	Residue	AA	Distance	Angle	Offset	Type	Ligand Atoms
1	246A	HIS	4.46	78.69	1.78	T	2870, 2877, 2879, 2883, 2884
2	246A	HIS	4.34	78.73	1.46	T	2872, 2879, 2880, 2882, 2884, 2887

Salt Bridges

Index	Residue	AA	Distance	Protein positive?	Ligand Group	Ligand Atoms
1	246A	HIS	4.28	✔	Carboxylate	2871, 2872

Hit 10 with 5R82

Hydrophobic Interactions

Index	Residue	AA	Distance	Ligand Atom	Protein Atom
1	132A	PRO	3.83	2887	1267
2	200A	ILE	3.45	2886	1885
3	202A	VAL	3.69	2877	1902
4	240A	GLU	3.81	2887	2294

Hydrogen Bonds

Index	Residue	AA	Distance H-A	Distance D-A	Donor Angle	Protein donor?	Sidechain	Donor Atom	Acceptor Atom
1	108A	PRO	2.26	2.87	168.93	✗	✗	2881 [Nam]	1049 [O2]
2	109A	GLY	2.97	3.82	140.75	✗	✗	2871 [Npl]	1056 [O2]
3	246A	HIS	2.77	3.35	116.72	✓	✓	2354 [N3]	2883 [O2]

Hit 11 with 5R82

Hydrogen Bonds

Index	Residue	AA	Distance H-A	Distance D-A	Donor Angle	Protein donor?	Sidechain	Donor Atom	Acceptor Atom
**1**	156B	ASN	2.09	2.96	146.77			3249 [Nam]	4485 [O3]
**2**	157B	ASP	2.56	3.36	138.91			4485 [O3]	3257 [O3]

π-Cation Interactions

Index	Residue	AA	Distance	Offset	Protein charged?	Ligand Group	Ligand Atoms
**1**	43B	TRP	4.46	1.74		quartamine	4486
**2**	43B	TRP	4.22	1		quartamine	4486
**3**	90B	TRP	4.2	1.21		quartamine	4486
**4**	90B	TRP	4.23	1.33		quartamine	4486
**5**	119B	TYR	4.37	0.94		quartamine	4486
**6**	205B	TRP	4.2	0.61		quartamine	4486
**7**	205B	TRP	4.45	1.58		quartamine	4486

Salt Bridges

Index	Residue	AA	Distance	Protein positive?	Ligand Group	Ligand Atoms
**1**	45B	ASP	4.77		Quartamine	4486

Hit 9 with 5R84

Hydrophobic Interactions

Index	Residue	AA	Distance	Ligand Atom	Protein Atom
1	108A	PRO	3.58	2895	1050
2	132A	PRO	3.25	2894	1267
3	200A	ILE	3.61	2893	1885
4	240A	GLU	3.52	2893	2294
5	249A	ILE	3.52	2889	2381

Hydrogen Bonds

Index	Residue	AA	Distance H-A	Distance D-A	Donor Angle	Protein donor?	Sidechain	Donor Atom	Acceptor Atom
1	109A	GLY	2.6	2.94	101.28	✘	✘	2897 [O3]	1056 [O2]
2	203A	ASN	2.63	3.26	120.5	✔	✔	1912 [Nam]	3]

π-Stacking

Index	Residue	AA	Distance	Angle	Offset	Type	Ligand Atoms
1	246A	HIS	4.45	80.64	1.85	T	2872, 2879, 2880, 2882, 2884, 2887

Salt Bridges

Index	Residue	AA	Distance	Protein positive?	Ligand Group	Ligand Atoms
1	246A	HIS	4.29	✔	Carboxylate	2871, 2872

Hit 10 with 5R84

Hydrophobic Interactions

Index	Residue	AA	Distance	Ligand Atom	Protein Atom
1	132A	PRO	3.78	2890	1267
2	240A	GLU	3.7	2890	2294

Hydrogen Bonds

Index	Residue	AA	Distance H-A	Distance D-A	Donor Angle	Protein donor?	Sidechain	Donor Atom	Acceptor Atom
1	108A	PRO	2.38	2.96	158.99	✗	✗	2881 [Nam]	1049 [O2]
2	109A	GLY	2.76	3.67	148.27	✗	✗	2871 [Npl]	1056 [O2]
3	246A	HIS	3.02	3.57	115.25	✓	✓	2354 [N3]	2883 [O2]

Hit 11 with 5R84

**Hydrophobic Interactions**									
**Index**	Residue	AA	Distance	Ligand Atom	Protein Atom				
**1**	106A	ILE	3.49	2889	1031				
**2**	110A	GLN	3.7	2889	1063				
**3**	151A	ASN	3.56	2875	1431				
**4**	151A	ASN	3.87	2881	1431				
**5**	153A	ASP	3.61	2879	1451				
**Hydrogen Bonds**									
**Index**	Residue	AA	Distance H-A	Distance D-A	Donor Angle	Protein donor?	Sidechain	Donor Atom	Acceptor Atom
**1**	158A	SER	3.06	3.81	136.49			1500	2872

								[O3]	[O2]
**2**	298A	ARG	2.38	2.86	107.97			2812 [Ng+]	2873 [O2]

**Halogen Bonds**							
**Index**	Residue	AA	Distance	Donor Angle	Acceptor Angle	Donor Atom	Acceptor Atom
**1**	295A	ASP	3.82	154.31	137.44	2870 [Cl]	2784 [O2]

Hit 9 with 5REC

Hydrophobic Interactions

Index	Residue	AA	Distance	Ligand Atom	Protein Atom
1	245A	ASP	3.64	2888	2340

<u>Hydrogen Bonds</u>

Index	Residue	AA	Distance H-A	Distance D-A	Donor Angle	Protein donor?	Sidechain	Donor Atom	Acceptor Atom
1	240A	GLU	3.13	3.89	132.32	✘	✔	2874 [Nox]	2296 [O2]
2	243A	THR	2.3	3.12	143.51	✔	✔	2320 [O3]	2897 [O3]
3	245A	ASP	3.36	4.07	134.23	✔	✔	2343 [O3]	2897 [O3]
4	246A	HIS	2.71	3.5	135.66	✔	✔	2354 [N3]	2870 [O2]

Hit 10 with 5REC

Hydrophobic Interactions

Index	Residue	AA	Distance	Ligand Atom	Protein Atom
1	108A	PRO	3.68	2877	1051
2	134A	PHE	3.7	2891	1289
3	134A	PHE	3.76	2878	1290

Hydrogen Bonds

Index	Residue	AA	Distance H-A	Distance D-A	Donor Angle	Protein donor?	Sidechain	Donor Atom	Acceptor Atom
1	132A	PRO	2.51	3.05	148.08	✗	✗	2881 [Nam]	1265 [O2]
2	240A	GLU	2	3.01	171.9	✗	✓	2871 [Npl]	2296 [O2]

Hit 11 with 5REC

Hydrophobic Interactions

**Index**	Residue	AA	Distance	Ligand Atom	Protein Atom
**1**	202A	VAL	3.52	2881	1902
**2**	202A	VAL	3.57	2875	1902
**3**	249A	ILE	3.72	2889	2380
**4**	249A	ILE	3.95	2886	2381
**5**	294A	PHE	3.5	2889	2775

Hydrogen Bonds

**Index**	Residue	AA	Distance H-A	Distance D-A	Donor Angle	Protein donor?	Sidechain	Donor Atom	Acceptor Atom
**1**	110A	GLN	3.56	3.98	108.4			2874 [Nam]	1065 [O2]

**2**	203A	ASN	3.64	4.08	108.87			1912 [Nam]	2872 [O2]
**3**	246A	HIS	2.36	3.19	138.73			2354 [N3]	2873 [O2]

6W63 with Hit 9

Hydrophobic Interactions

Index	Residue	AA	Distance	Ligand Atom	Protein Atom
1	41A	HIS	3.88	2900	378

Hydrogen Bonds

Index	Residue	AA	Distance H-A	Distance D-A	Donor Angle	Protein donor?	Sidechain	Donor Atom	Acceptor Atom
1	54A	TYR	3.36	4.07	127.86	✘	✔	2883 [Nox]	505 [O3]
2	189A	GLN	2.19	2.93	144.35	✔	✔	1795 [Nam]	2906 [O3]

6W63 with Hit 10

Hydrophobic Interactions

Index	Residue	AA	Distance	Ligand Atom	Protein Atom
**1**	104A	VAL	3.75	2899	1006
**2**	106A	ILE	3.62	2896	1031
**3**	110A	GLN	3.88	2895	1063
**4**	110A	GLN	3.99	2885	1062

Hydrogen Bonds

Index	Residue	AA	Distance H-A	Distance D-A	Donor Angle	Protein donor?	Sidechain	Donor Atom	Acceptor Atom
**1**	110A	GLN	3.19	4.05	176.21			1066 [Nam]	2890 [Nam]
**2**	111A	THR	2.67	3.49	158.87			1070 [Nam]	2880 [Npl]
**3**	111A	THR	2.86	3.30	106.32			2880 [Npl]	1075 [O3]

Halogen Bonds

Index	Residue	AA	Distance	Donor Angle	Acceptor Angle	Donor Atom	Acceptor Atom
1	105A	ARG	3.22	136.94	112.61	2898 [Cl]	1011 [O2]

6W63 with Hit 11

Hydrophobic Interactions

Index	Residue	AA	Distance	Ligand Atom	Protein Atom
**1**	41A	HIS	3.74	2898	378
**2**	49A	MET	3.69	2893	451
**3**	165A	MET	3.78	2891	1574
**4**	189A	GLN	3.93	2887	1791

Halogen Bonds

Index	Residue	AA	Distance	Donor Angle	Acceptor Angle	Donor Atom	Acceptor Atom
**1**	145A	CYS	3.68	146.43	141.93	2879 [Cl]	1386 [S3]
